# Correlation of Hepatic Steatosis Among Cohabitants Using Hounsfield Unit From Coronary Computed Tomography

**DOI:** 10.7759/cureus.17834

**Published:** 2021-09-08

**Authors:** Zafar Ali, Ibrahim M Saeed, Kevin A Bybee, Randall Thompson, James H O’Keefe, Muhammad Shafiq, Lyla Saeed, Yousaf Zafar, Kevin F Kennedy, Leen Al-Sayyed

**Affiliations:** 1 Internal Medicine, The University of Kansas Medical Center, Kansas City, USA; 2 Cardiovascular Disease, Virginia Heart, Falls Church, USA; 3 Cardiovascular Disease, Saint Luke's Mid America Heart Institute, Kansas City, USA; 4 Internal Medicine, University of Missouri Kansas City School of Medicine, Kansas City, USA; 5 Internal Medicine, Naples Community Healthcare, Naples, USA; 6 Biostatistics, Saint Luke's Mid America Heart Institute, Kansas City, USA; 7 Gastroenterology and Hepatology, University of Missouri Kansas City School of Medicine, Kansas City, USA

**Keywords:** risk factor, hounsfield unit, hepatic steatosis, computed tomography scan, cohabitants

## Abstract

Background

Individuals living in the same household are exposed to common risk factors. We hypothesized that living with someone who has fatty liver disease increases the risk of having the same disease.

Methods

This was a retrospective study that included pairs of men and women who shared the same residential addresses, underwent screening non-contrast computed tomography for coronary calcium scoring and had Hounsfield Unit density for liver and spleen in the field of view available for measurement. The primary goal was to determine the association between hepatic steatosis and living in the same household. Secondary end-points compared to body mass index, triglyceride levels, type 2 diabetes mellitus (T2DM) and hypertension.

Results

Out of 1,362 cohabitant pairs, there were 202 couples with either the male or female having hepatic steatosis and 10 cohabitant pairs with both the male and female having hepatic steatosis. In 1,150 cohabitant pairs out of 1,362, neither man nor woman had hepatic steatosis. Pearson correlation coefficient (r) for hepatic steatosis between cohabitant pairs was 0.122 (p-value: < 0.001), suggesting that no correlation was found. Elevated triglyceride levels were prevalent among cohabitant pairs with hepatic steatosis, when compared to pairs without hepatic steatosis (p-value < 0.05). Female gender and having a diagnosis of hepatic steatosis also showed a strong association with higher body mass index, T2DM and hypertension (p-value < 0.05).

Conclusion

Despite the assumption of exposure to similar environmental factors, our results did not show any correlation of hepatic steatosis among the cohabitants.

## Introduction

Hepatic steatosis or fatty liver is a broad term applied to a wide spectrum, characterized by triglyceride accumulation within the hepatocytes [1]. Non-alcoholic fatty liver disease (NAFLD), also known as primary fatty liver disease, is characterized by the presence of hepatic steatosis in the absence of any apparent cause [2]. Secondary causes of hepatic steatosis include alcohol use, viral hepatitis, genetic disorders, use of certain medications, endocrine disorders and type of nutrition [2-4]. Of all types, NAFLD and alcoholic fatty liver disease are the most common causes of hepatic steatosis [5,6].

Patients with NAFLD commonly have obesity, essential hypertension, type 2 diabetes mellitus (T2DM) and hyperlipidemia, which are all components of the metabolic syndrome [7-9]. NAFLD also increases the risk of cardiovascular disease, T2DM and chronic kidney disease [10]. It has been shown as well that NAFLD is independently associated with increased risk for cardiovascular disease after controlling for age, sex, T2DM, obesity, smoking history and family history of coronary artery disease (CAD) [11]. Thus, NAFLD is in many ways a multisystem disease.

Given increasing prevalence and worse outcomes, there has been growing interest in identifying potential risk factors and remedies for NAFLD. Many risk factors have been identified for NAFLD, but ones of interest are the modifiable risk factors, which include; eating habits, exercise, obesity, hypertension, glycemic control and tobacco smoking [12-15]. Some studies have demonstrated that members of the same household are exposed to or share common risk factors [16-18]. This idea led to our quest of knowing whether living together with someone known to have fatty liver disease increases one’s risk of the disease as well.

We hypothesized that adults living in the same household frequently have concordance of fatty liver disease due to the exposure to similar environmental factors. The objective of our study was to assess the association of fatty liver among cohabitants, utilizing computed tomography (CT) scan performed for coronary artery calcium scoring.

## Materials and methods

Approval for this study was provided by Saint Luke’s Hospital Institutional Review Board (IRB), under the protocol number 18-091. Saint Luke's Hospital is located in Kansas City, MO, USA. 

Study design

This study was designed as a retrospective cross-sectional study, based on chart review. All adult patients who received non-contrast cardiac CT scans for coronary calcium scoring between January 1st, 2000 to June 30th, 2018 were screened for selection (n = 101,852). Cohabitants were defined as pairs of men and women who shared the same residential addresses. These individuals were the focus of the study. In study hospital, coronary artery calcium scores are available for patient self-referral at a modest price. In fact, many couples have their scans together, and there are sometimes specials for couples during February (Heart Month).

Participants

Inclusion criteria included cohabitants, who were 18 years of age & older and had Hounsfield Unit (HU) for liver and spleen available via screening non-contrast X-ray CT scans done for coronary calcium scoring. Cohabitants who had ≥ 5 years age difference were excluded.

Intervention

A liver density of < 40 HU or ≥ 10 HU less than splenic density was used as the definition of hepatic steatosis in this study. The coronary CT scans were interpreted by experienced cardiologists specializing in coronary CT imaging. As part of the protocol, HU unit densities for liver and spleen were measured by trained radiology technologists by drawing regions of interest within the liver and spleen, avoiding vessels and cysts, and these measurements were reviewed by the interpreting cardiologist.

Outcomes

The primary end-point was to identify the association between hepatic steatosis and living at the same household/residence. Secondary end-points included comparison of body mass index (BMI), triglyceride levels, T2DM and hypertension history between the cohabitant pairs with hepatic steatosis and cohabitant pairs without hepatic steatosis.

Statistical analysis

Pearson correlation was used to determine the association between hepatic steatosis among subjects who were living together, the primary endpoint. Student’s t-test was used to compare BMI between cohabitant pairs with hepatic steatosis and cohabitant pairs without hepatic steatosis. Chi-square test or fisher exact test was used to compare triglyceride levels, T2DM and hypertension history between the cohabitant pairs with hepatic steatosis and cohabitant pairs without hepatic steatosis. Alpha criterion for all statistical tests was set at 0.05. All analyses were performed with SAS 9.4 (Cary, NC).

## Results

**Figure 1:**
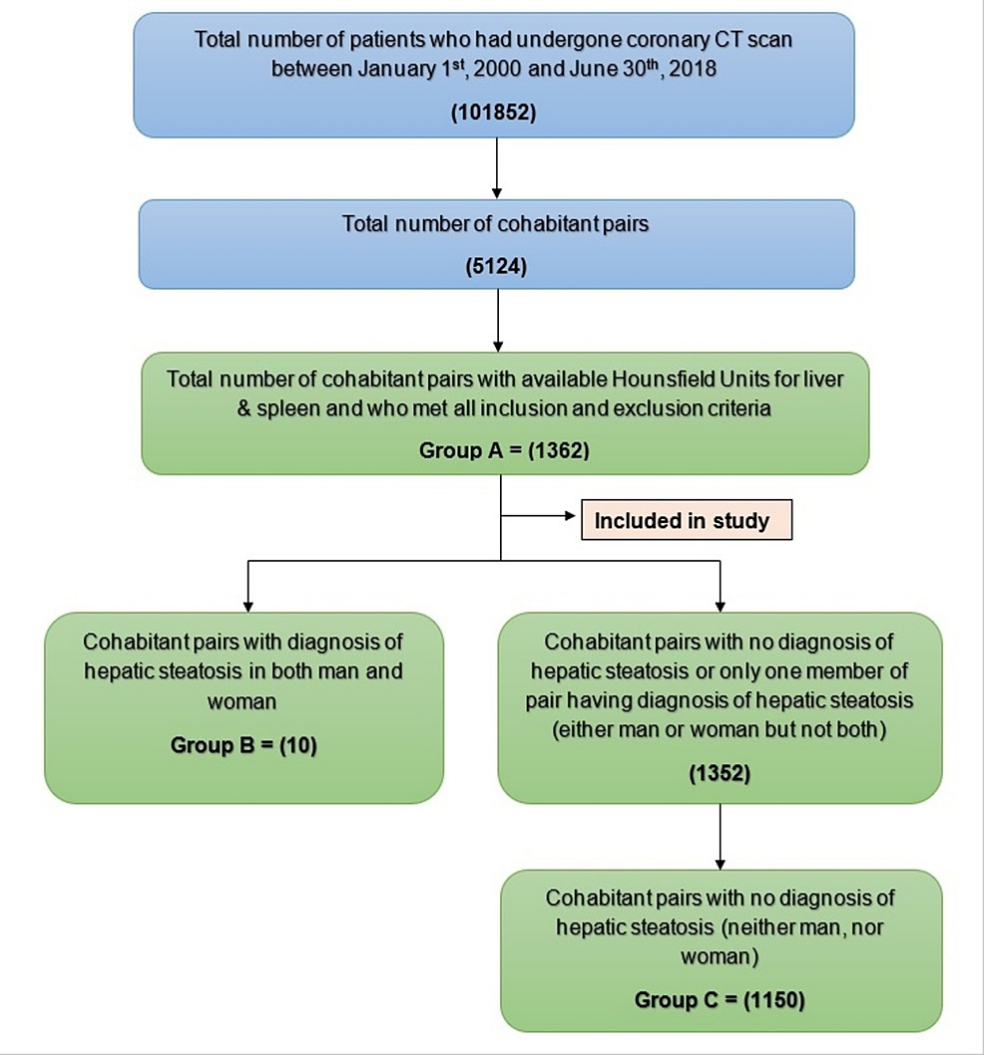
Cohabitant pairs selection flowchart. Out of 101,852 identified patients, 1,362 patients (Group A) were included in the study based on inclusion and exclusion criteria. Group A was further divided into two sub-groups whether the cohabitant pair had hepatic steatosis (B) or not (C).

The total number of coronary CT scans done between January 1, 2000 to June 30th, 2018 was 101,852. Of those, 1,362 cohabitant pairs had HU for spleen and liver available within the field of view of the CT scan and were included in the study (group A). Out of 1,362 cohabitant pairs, there were 202 couples with either the male or female having hepatic steatosis and 10 cohabitant pairs with both the male and female having hepatic steatosis (group B). In 1,150 cohabitant pairs out of 1,362, neither man nor woman had hepatic steatosis (group C) as illustrated in Figure 1.

Age and gender comparison of groups B and C are presented in Table 1.

**Table 1:** Age and gender comparison between cohabitants with and without hepatic steatosis.

	Age of cohabitants with hepatic steatosis (in years) Group B (n = 10 pairs)	Age of cohabitants without hepatic steatosis (in years) Group C (n = 1,150 pairs)	p-value
Male cohabitants	55.80 ± 6.97	58.75 ± 9.51	0.337
Female cohabitants	54.30 ± 7.83	57.69± 9.41	0.277

**Figure 2:**
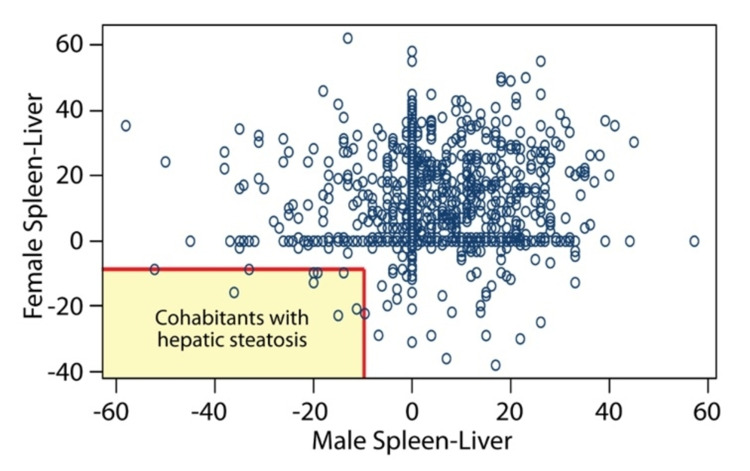
Correlation of hepatic steatosis between cohabitant pairs. Hounsfield units for the male partner are on x-axis and for female partner on y-axis. This Pearson correlation plot doesn't show any linear pattern to suggest association between hepatic steatosis and cohabitation. There were only 10 pairs who had hepatic steatosis and are highlighted by the red-square in the left lower corner.

Pearson correlation coefficient (r) for hepatic steatosis in Group A, between cohabitant pairs, was 0.122 (p-value: < 0.001). As this r is very close to zero, it suggests that no correlation was noted between living at the same household and hepatic steatosis; hence failing to reject the null hypothesis (Figure 2). Correlation of hepatic steatosis among age controlled non-cohabitant pairs was r = 0.018 (p-value = 0.051).

Among the secondary end-points; it was noted that elevated triglyceride levels were more prevalent among cohabitant pairs with hepatic steatosis (Group B), when compared to pairs without hepatic steatosis (Group C) with p-value < 0.05. Only female partners of cohabitant pairs with hepatic steatosis (Group B) were noted to have statistically significant higher BMI, more prevalent T2DM and hypertension when compared to their counterparts in the group without hepatic steatosis (Group C), as shown in Table 2.

**Table 2:** Secondary end-points comparison between cohabitants with and without hepatic steatosis. BMI: body mass index; TG: triglycerides; T2DM: type 2 diabetes mellitus.

	Cohabitants with hepatic steatosis Group B (n = 10 pairs)	Cohabitants without hepatic steatosis Group C (n = 1150 pairs)	p-value
BMI of male cohabitants	30.51 ± 7.00	29.54 ± 10.51	0.302
BMI of female cohabitants	34.26 ± 9.70	27.60 ± 7.26	0.020
Elevated TG in male cohabitants	5 (50.0%)	260 (22.6%)	0.039
Elevated TG in female cohabitants	5 (50.0%)	208 (18.1%)	0.009
T2DM in male cohabitants	2 (20.0%)	102 (8.9%)	0.219
T2DM in female cohabitants	4 (40.0%)	60 (5.2%)	<0.001
Hypertension in male cohabitants	4 (40%)	429 (37.3%)	0.940
Hypertension in female cohabitants	8 (80%)	321 (33.8%)	<0.001

## Discussion

To our knowledge, this is the first study that has looked into the correlation of hepatic steatosis among cohabitants. Living in the same household intuitively increases the likelihood of exposure to the same risk factors such as dietary habits and exercise [16,18-20]. Therefore, we hypothesized that if someone is diagnosed with hepatic steatosis, it would increase the likelihood of a similar diagnosis among household members such as spouses/domestic partners. Should this have been true, it might have led to a recommendation for screening family members for timely diagnosis and management of hepatic steatosis. Despite ample epidemiological evidence of concordance of shared dietary and other lifestyle habits among cohabitants as discussed above, it was surprising to us that our study did not find an association between cohabitants sharing the common diagnosis of hepatic steatosis (including NAFLD) [16, 18-20]. Cohabitant pairs with hepatic steatosis were noted to have a higher prevalence of elevated triglyceride levels. Female gender and having a diagnosis of hepatic steatosis also showed a strong association with higher BMI, T2DM and hypertension.

The sensitivity of using CT attenuation of the liver for the establishment of fatty liver disease has been previously established [21,22]. Some studies have also established a high correlation of CT scan attenuation to fibrosis of liver established by biopsy [23]. CT coronary artery calcium scoring is a commonly performed test for the presence of CAD. At our center, hepatic and splenic HU densities are routinely measured when patients undergo coronary artery calcium scoring. This approach allows clinicians to use the cardiac CT as a cost-effective tool for the detection of fatty liver disease. Patients who are identified as having hepatic steatosis are usually referred for hepatology consultation and to undergo further investigations comprising of hepatic serologies and noninvasive tests such as magnetic resonance elastography. If needed, liver biopsy is also pursued. Many of these patients are also referred to our Cardio-Wellness Clinic and our Cardio-Metabolic Center to help initiate lifestyle modification.

NAFLD is common and now has an estimated global prevalence of approximately 25% and has become an important public health concern [24]. Yet, our knowledge of NAFLD is incomplete and exact pathogenesis is yet to be elucidated. Prior perception of NAFLD being a simple byproduct of environmental factors such as diet, does not help explain why lifestyle modifications have no linear effect on NAFLD outcomes. It is not known why some individuals develop progression of fatty liver disease to irreversible stages. Our study was one step in further understanding the unknown of NAFLD.

Though, we could not show any significant correlation of hepatic steatosis among the cohabitants, there are certain limitations to our study. This study was single-center, non-randomized and observational. Given the observational nature of the study, unmeasured cofounding variables such as dietary habits, alcohol consumption, frequency of exercise and abdominal girth of each participant could not be controlled for in our analysis. The duration of cohabitation was unknown. Also, there were only 10 cohabitant pairs with both the male and female having hepatic steatosis.

## Conclusions

Despite the assumption of exposure to similar environmental factors, our results did not show any correlation of hepatic steatosis among cohabitants. The study did demonstrate the potential for X-ray coronary calcium scoring to be used as a cost-effective screening tool for hepatic steatosis in a population being screened for coronary artery disease. This approach might lead to earlier recognition and interventions to prevent the progression of hepatic steatosis to fibrosis and liver cirrhosis.
